# Impact of pre-exposure prophylaxis uptake among gay, bisexual, and other men who have sex with men in urban centers in Brazil: a modeling study

**DOI:** 10.1186/s12889-023-15994-0

**Published:** 2023-06-13

**Authors:** Paula M. Luz, Vijeta Deshpande, Pooyan Kazemian, Justine A. Scott, Fatma M. Shebl, Hailey Spaeth, Cristina Pimenta, Madeline Stern, Gerson Pereira, Claudio J. Struchiner, Beatriz Grinsztejn, Valdilea G. Veloso, Kenneth A. Freedberg

**Affiliations:** 1 Instituto Nacional de Infectologia Evandro Chagas, Fundação Oswaldo Cruz, Avenida Brasil 4365, Rio de Janeiro, 21040-360 Brazil; 2 Medical Practice Evaluation Center, Massachusetts General Hospital, 100 Cambridge Street, Suite 1684, Boston, MA 02114 USA; 3 Department of Operations, Weatherhead School of Management, Case Western Reserve University, 11119 Bellflower Road, Cleveland, OH 44106 USA; 4 Ministry of Health of Brazil, SRTVN Quadra 701, Lote D, Edifício PO700, 5º Andar, Brasília/DFBrasilia, 70719-040 Brazil; 5 Escola de Matemática Aplicada, Fundação Getúlio Vargas, Rio de Janeiro, Brazil; 6 Harvard Medical School, 25 Shattuck Street, Boston, MA 02115 USA; 7 Harvard University Center for AIDS Research, Harvard Medical School, 42 Church Street, Cambridge, MA 02138 USA; 8 Division of General Internal Medicine, Massachusetts General Hospital, 55 Fruit Street, Boston, MA 02114 USA; 9 Division of Infectious Diseases, Massachusetts General Hospital, 55 Fruit Street, Boston, MA 02114 USA; 10 Department of Health Policy and Management, Harvard School of Public Health, 677 Huntington Ave, Boston, MA 02115 USA

**Keywords:** Pre-exposure prophylaxis (PrEP), HIV prevention, Men who have sex with men, Modeling, Latin America and the Caribbean, Key and vulnerable populations

## Abstract

**Background:**

Men who have sex with men (MSM) in Brazil remain disproportionately affected by HIV. We estimated the potential incidence reduction by five years with increased uptake of publicly-funded, daily, oral tenofovir/emtricitabine (TDF/FTC) for HIV pre-exposure prophylaxis (PrEP) among MSM using the Cost Effectiveness of Preventing AIDS Complications microsimulation model. We used national data, local studies, and literature to inform model parameters for three cities: Rio de Janeiro, Salvador, and Manaus.

**Results:**

In Rio de Janero, a PrEP intervention achieving 10% uptake within 60 months would decrease incidence by 2.3% whereas achieving 60% uptake within 24 months would decrease incidence by 29.7%; results were similar for Salvador and Manaus. In sensitivity analyses, decreasing mean age at PrEP initiation from 33 to 21 years increased incidence reduction by 34%; a discontinuation rate of 25% per year decreased it by 12%.

**Conclusion:**

Targeting PrEP to young MSM and minimizing discontinuation could substantially increase PrEP’s impact.

**Supplementary Information:**

The online version contains supplementary material available at 10.1186/s12889-023-15994-0.

## Background

In Brazil, men who have sex with men (MSM) continue to be at substantial risk of HIV acquisition, with nationally representative, respondent-driven sampling studies showing an increasing HIV prevalence, from 14.2% in 2009 to 18.4% in 2016; this is more than 20 times the prevalence in the general population [1, 2]. HIV incidence among MSM varies across the country, ranging from 0.9 to 1.5/100 person-years (PY) in Recife and Curitiba [3], to 5.0/100PY in Rio de Janeiro and São Paulo [4], and 7.4/100PY in the most recent study conducted in Rio de Janeiro from 2018 to 2020 [5]. Incidence among MSM has continued to increase over the past years, accounting for 72% of cases in 2021 compared to 65% in 2015 [6].

There is strong evidence from trials and observational studies that oral pre-exposure prophylaxis (PrEP) is effective in preventing HIV in MSM [7–9]. Since 2013, the World Health Organization has issued guidelines on the use of antiretrovirals for HIV prevention. The latest guideline addressing daily oral antiretrovirals suggests that this prevention option should be offered to people at substantial risk of acquiring HIV, defined as HIV incidence higher than 2/100PY [10]. A model-based analysis of oral PrEP use among adults at substantial risk of HIV in sub-Saharan Africa showed that it can reduce HIV incidence by 44% over a 5-year time horizon and that it would be a cost-effective strategy in settings where HIV prevalence is higher than 2% among adults [11]. Such modeling studies can project the population-level expected impact of implementing PrEP, particularly when setting-specific data are used to inform the analysis. We are not aware of any study focused on the impact of PrEP for Brazil.

In December 2017, to address the continuing HIV epidemic in Brazil, the Brazilian National Health System approved daily oral PrEP with tenofovir/emtricitabine (TDF/FTC) for select populations. This included eligible MSM engaging in condomless receptive anal intercourse in the previous six months and/or having symptoms or diagnosis of sexually transmitted infections in the previous six months and/or reporting repeated use of post-exposure prophylaxis in the prior 12 months [12]. PrEP uptake among eligible MSM varied considerably in 2018, the program’s first year, reaching 8% in Rio de Janeiro and 10% in São Paulo, but less than 5% in half of the cities evaluated in a recent study [13]. Targets for PrEP uptake globally among those at risk of HIV have not been uniformly adopted, varying as a function of key populations and risk levels [14].

Simulation modeling as a methodology has been increasingly utilized in health care research over the past several decades, to project longer-term outcomes from both observational studies and randomized trials [15, 16]. For PrEP specifically, we and others have used simulation modeling to assess the impact and cost-effectiveness of different modes of PrEP in the United States, South Africa, India, and Brazil [11, 17–19]. Our objective in this study, now that the PrEP rollout is underway, was to model the impact of increased uptake of PrEP among eligible MSM in Brazil.

## Methods

### Analytic overview

We used the Cost Effectiveness of Preventing AIDS Complications (CEPAC) model, a widely published agent-based state-transition microsimulation model of HIV disease and treatment, to project the clinical impact of daily oral TDF/FTC-based PrEP [17–20]. In a prior analysis, we estimated that providing PrEP to MSM and transgender women at substantial risk of acquiring HIV would be a cost-effective strategy from the perspective of Brazil’s national health system [19]. We simulated a cohort of adult (age ≥ 18 years) MSM without HIV at substantial risk of HIV infection. As mentioned above, though PrEP is currently offered in Brazil, uptake is low and heterogeneous across cities. As such, to standardize comparison within and across cities, we compared the impact of various levels of PrEP uptake with the counterfactual scenario of no PrEP use. To describe different PrEP uptake scenarios, we focused on two parameters: (1) Maximum uptake, which represents the maximum PrEP coverage among MSM in a particular city, and (2) Time in months required to reach maximum uptake, after which uptake is maintained at that level. We explored a wide range of assumptions regarding maximum uptake (10 to 60%) and time required to achieve that maximum level (24 to 60 months). Outcomes included number of HIV infections, number of averted infections, and percent incidence reduction due to PrEP. In sensitivity analyses, we varied key parameters, including PrEP adherence, HIV testing frequency, and age at PrEP initiation. Additionally, we considered the possibility of “discontinuation” or abandonment of the PrEP program.

### CEPAC model

#### PrEP and HIV testing

PrEP is simulated as a reduction in the probability of HIV infection. This reduction in infection risk is the direct individual benefit of PrEP, applied to individuals taking PrEP, for the duration that PrEP is used. Each averted infection due to this direct individual benefit may subsequently prevent further HIV transmissions. This reduction in later, secondary transmissions, is the indirect community benefit of PrEP, which is conferred upon all individuals in the cohort regardless of whether they are taking PrEP (see Additional file 1 & 2 for additional details). Simulated individuals can be tested for HIV in two ways: through background HIV testing, based on current testing rates in Brazil, or through regular testing as part of a PrEP intervention.

#### HIV acquisition and transmission

Susceptible individuals are exposed to an age dependent infection risk. We simulated HIV transmission in a cohort of adult MSM with and without a PrEP intervention using a previously validated methodology [18, 20]. The benefits of a PrEP intervention are two-fold: 1) direct individual benefit and 2) indirect community benefit, both of which imply a reduction in the incidence of HIV for the simulated population (Additional files 1 and 2). The direct individual benefit is a reduction in the risk of HIV infection that is experienced by the individual taking PrEP, for the duration that PrEP is used, and is a function of drug efficacy and adherence. The indirect community benefit of PrEP is the benefit due to prevented HIV infections that otherwise would have occurred, which leads to having fewer infected individuals in the community, over the simulation horizon. This indirect community benefit is experienced by the entire cohort regardless of PrEP uptake status. Because we focus on high risk MSM, we account only for HIV transmissions within this population, from individuals with HIV to susceptible individuals at increased risk of acquiring HIV, when calculating the community benefit of PrEP (Additional file 2).

### Model inputs

#### The HIV epidemic in selected cities

The HIV epidemic in Brazil is concentrated in large urban centers, with 51% of all infections reported in the Southeast region of the country [6]. Because the HIV epidemic varies regionally in Brazil in terms of prevalence, incidence, sexual behavior, and HIV testing frequency [21], we modeled three cities with distinct epidemiologic profiles. These cities represent three of the five regions of Brazil and are the largest urban centers in their respective states: Rio de Janeiro (capital of the state of Rio de Janeiro, in the Southeast region, population 6.7 million), Salvador (capital of Bahia, Northeast, 2.9 million), and Manaus (capital of Amazonas, North, 2.2 million) [22]. We focused on these cities for multiple reasons: 1) these cities have the largest HIV epidemics of their regions, 2) well-designed epidemiologic studies have been conducted in these cities which have provided good data to inform model parameters, 3) though PrEP is available in Brazil, not all municipalities currently provide PrEP and these cities have well-established HIV prevention centers offering PrEP to eligible populations.

To model the HIV epidemic and to describe the characteristics of the target population eligible for PrEP in these cities, we used the best available Brazilian national behavioral surveillance surveys, conducted in 2009 and 2016, as well as online surveys conducted between 2016 and 2018, all focused on MSM. The national behavioral surveillance studies [1, 2], funded by the Department of HIV/AIDS, Tuberculosis, Viral Hepatitis and STI of the Ministry of Health, were conducted in ten cities (three of which are the cities considered in the present study) to inform on the HIV prevalence among MSM in Brazil. HIV prevalence estimates from these studies were used to inform city- and age-specific incidence rates, described below. Results from online surveys that included over 16,000 MSM informed the mean age of the cohort (ranged from 25–30 years), the reported frequency of ever having been tested for HIV (83%, 83% and 75% in Rio de Janeiro, Salvador and Manaus), and the reported frequency of high risk sexual behavior such as condomless receptive anal sex in the past six months (42.1%, 40.2%, and 39.9% in Rio de Janeiro, Salvador, and Manaus) (Table 1) [21]. Using these demographic and epidemiological data, we used previously published methodology [13, 23] to infer the size of the HIV-infected and uninfected population (Table 1) from the estimated size of the male population of each city, as well as the proportion who engage in sex with other men, who were sexually active in the prior six months, who engage in unprotected anal sex with men, and who are not HIV infected such that the target population represents the fraction of MSM that would be eligible for PrEP.

**Table 1:** Select model input parameters

City specific model inputs	Rio de Janeiro	Salvador	Manaus	Reference	
**Cohort characteristics**					
Age, mean years (SD)	30 (9.6)	29 (8.1)	25 (5.9)	[21]	
Size of HIV infected population	16,999	3,926	2,828	[1, 21]	
Size of HIV uninfected population	94,104	41,728	45,937	[1, 21]	
Initial CD4 count for chronic cases, mean cells/μl (SD)	492 (320)	451 (299)	420 (281)	Estimated from national laboratory and treatment datasets for each city	
**Age-specific HIV incidence rate (per 100PY)**					
16–18 years	4.26	2.43	1.39	Estimated based on [1–4], see Additional file 3 for details	
18–24 years	4.44	2.53	1.44		
25–29 years	4.38	2.50	1.43		
30–39 years	3.75	2.14	1.22		
40–45 years	1.90	1.08	0.62		
46–50 years	1.00	0.57	0.32		
51–55 years	0.51	0.29	0.17		
> 55 years	0.27	0.16	0.09		
**HIV testing characteristics**					
Background HIV testing rate (per 100PY)	13.0	11.0	8.7	[21]	
**PrEP characteristics**					
Adherence, % (CI)	66.0 (62.7–69.2)	64.9 (59.0–70.8)	68.5 (64.3–72.7)	*ImPrEP*	
**Other model inputs, not city specific**				**All cities**	
**PrEP characteristics**					
Efficacy (%)				96	[24, 25]
Uptake (%)				10—60	Assumption
Time to reach maximum uptake (months)				12—48	Assumption
Frequency of HIV testing on PrEP, tests/year				4	Assumption
**Clinical characteristics post HIV infection**					
Initial CD4 count for acute cases, mean cells/μl (SD)				559 (236)	[26, 27]
Initial suppression, %					[28–30]
First-line (DTG + TDF + FTC)				88	
Second-line (DRV/r + 2 NRTIs)				73	
Third-line (DTG + PI + 2 NRTIs)				88	
Rate of virologic failure, instances/100 PM					[31, 32]
First-line (DTG + TDF + FTC)				0.35	
Second-line (DRV/r + 2 NRTIs)				0.93	
Third-line (DTG + PI + 2 NRTIs)				0.54	
Monthly CD4 increase on suppressive ART, cells/µL, mean (SD)					[33]
First month				80.4 (30)	
After first month				4.2 (1.5)	

*SD* Standard deviation, *HIV* Human immunodeficiency virus, *PY* Person-years, *PrEP* Pre-exposure prophylaxis, *CI* Confidence interval, *ART* Antiretroviral treatment, *DTG* Dolutegravir, *TDF* Tenofovir disoproxil fumarate, *FTC* Emtricitabine, *DRV/r* Darunavir/ritonavir, *NRTIs* Nucleoside reverse transcriptase inhibitor, *PI* Protease inhibitor, *PM* Person-months

Using national data from the Ministry of Health [34] of MSM who initiated treatment between 2016 and 2018, we characterized the immunological profile of MSM initiating care. Participants sought care earlier in Rio de Janeiro, with mean CD4 count of 492 (standard deviation [SD] 320) cells/mm^3^ compared to 451 (SD 299) cells/mm^3^ and 420 (SD 281) cells/mm^3^ in Salvador and Manaus, respectively.

#### Age-specific HIV incidence

Because HIV incidence rates among MSM were not available for each of the three cities, we used data from multiple sources to generate age specific HIV incidence rates for eight age groups (Table 1). From published incidence rates from iPrEx [4], we inferred a reduction of 77% in incidence rate after age 40. From HIV prevalence estimates [1, 2], we back calculated the incidence rate assuming a simple susceptible/infected (SI) model, such that HIV prevalence would reach the assumed prevalence at a mean age of ~ 30 years. We fitted a separate Gaussian curve for each city over the incidence estimates and extracted the incidence rate estimates for the eight age strata from the fitted curve (Additional file 3).

#### PrEP characteristics

In line with a previous analysis [19] and prior literature [35, 36], we define PrEP effectiveness as the composite of drug efficacy in a highly adherent trial-based setting and adherence to daily oral PrEP pills. As such, we modeled PrEP effectiveness, or the reduction in the risk of HIV infection to individuals taking PrEP (the direct benefit), as the product of efficacy and adherence. PrEP efficacy in those adherent was assumed at 96% which is PrEP’s estimated efficacy among MSM taking 4 doses per week as derived by a pharmacokinetic model and recently confirmed with directly observed therapy [24, 25]. PrEP adherence, defined as the proportion of individuals that took 4 (or more) doses per week [37], was informed by *ImPrEP*, a transnational implementation project in Latin America on the feasibility, acceptability, and economic impact of PrEP among key populations in Brazil, Mexico, and Peru [38]. We used estimates of the medication possession ratio of *ImPrEP* for participants from each of the three cities to estimate the proportion of adherent individuals during a participant’s first year of PrEP. We estimated the average adherence to be 66.0% in Rio de Janeiro, 64.9% in Salvador, and 68.5% in Manaus (Table 1). HIV testing as part of the PrEP program was modeled at a frequency of four tests per year in the base case, as per recommended guidelines for PrEP provision in Brazil [12].

By design of our study, PrEP uptake varied in regard to its maximum value (10 to 60%) and time required to achieve that maximum level (24 to 60 months). Though uptake in *PrEP Brasil*, a demonstration project assessing PrEP delivery in three well established reference centers for HIV prevention and care in Rio de Janeiro and São Paulo from 2014 to 2015 was 60.9% [39], it was much lower in the context of PrEP implementation through the National Health System, reaching only 8% in Rio de Janeiro in 2018 (Salvador: 3.1%, Manaus: 6.9%) [13]. We assumed that the maximum uptake level, once attained, would be maintained for the remainder of the time horizon. That is, once the individual is assumed to uptake PrEP, then he remains in use for the length of the time horizon. This assumption is relaxed in sensitivity analyses when we consider discontinuation from the PrEP intervention.

### Analysis

#### Main outcomes

For each city, we compared the projected number of HIV infections at five years, with PrEP (PrEP scenario) and without PrEP (No PrEP scenario). For each PrEP intervention, we calculated number of averted HIV infections and percent reduction in HIV incidence relative to No PrEP.

#### Sensitivity analyses

We assessed parameter uncertainty in deterministic one-way and two-way sensitivity analyses. For these, we assumed 30% maximum uptake achieved within 36 months. In one way sensitivity analyses, we varied adherence to PrEP, discontinuation from the PrEP program, HIV testing frequency as part of the PrEP intervention, and mean age of PrEP initiation. Parameter ranges were from local studies. As described above, *ImPrEP* informed the point estimate for adherence, with range defined as the 95% confidence interval for the medication possession ratio during a participant’s first year of PrEP use. Discontinuation rates were also from *ImPrEP* and estimated as 16.9% in Rio de Janeiro (95% confidence interval (CI) 14.6–19.4), Salvador 17.6% (95%CI 13.7–22.7), Manaus 16.2% (95%CI 13.3–19.8) per year. We considered testing frequencies from monthly to every six months for individuals in the PrEP program. We considered a mean age of PrEP initiation from 21 to 33 years (Table 1). In two way sensitivity analyses, we examined the joint effect of varying PrEP adherence and discontinuation rates.

## Results

### Base case

**Fig. 1:**
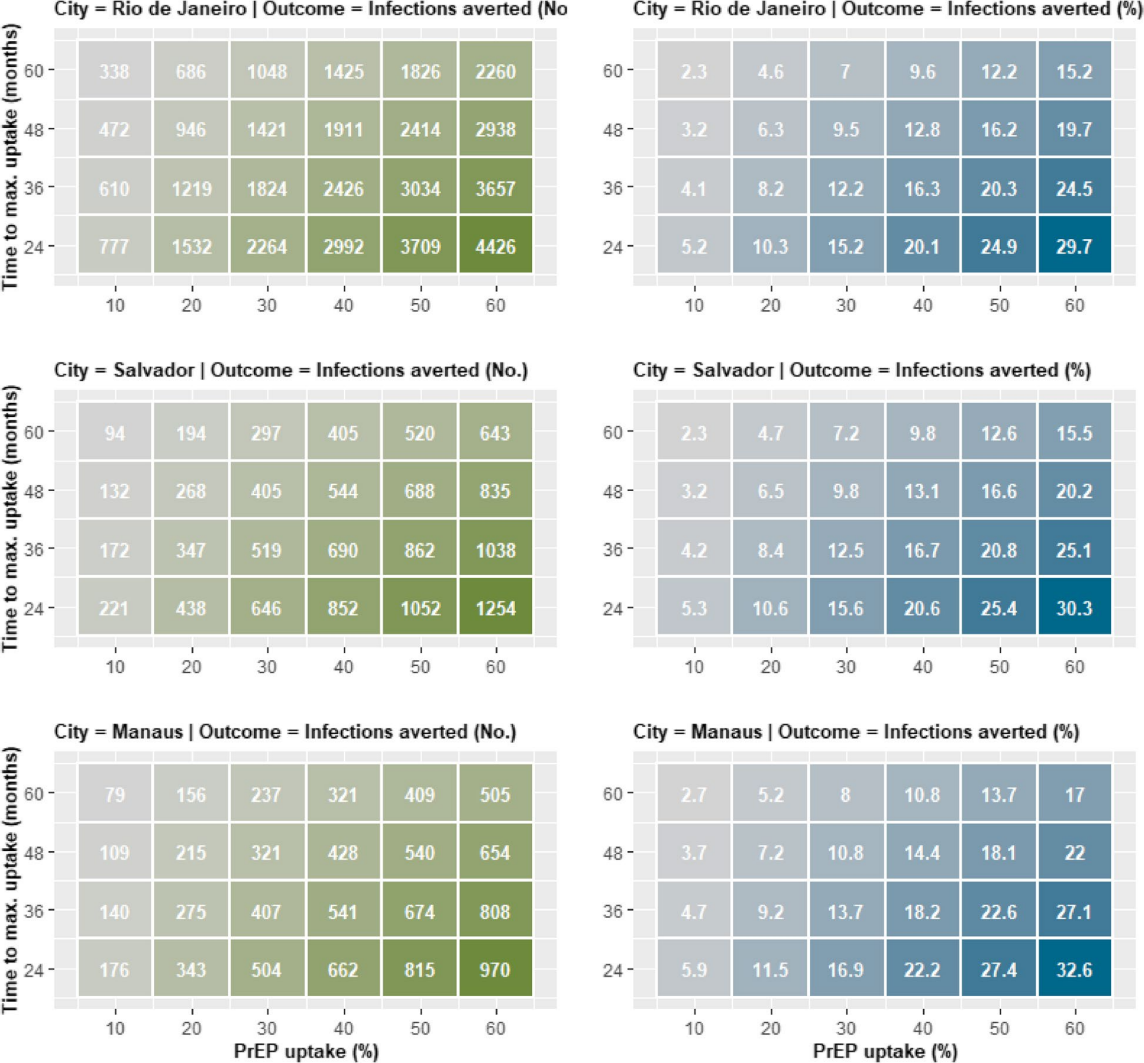
Five-year HIV infections averted (left side in green) and percent of infections averted (right side in blue) as a function of PrEP uptake (horizontal axis) and time to uptake (vertical axis). Darker colors indicate higher number of infections averted or percent reduction. Each PrEP intervention strategy is defined by the maximum uptake level of PrEP (x-axis, from 10 to 60%) and time to reach the maximum uptake (y-axis, from 24 to 60 months). Top plot: Rio de Janeiro, middle: Salvador, bottom: Manaus

The projected number of HIV infections at five years without PrEP (No PrEP scenario) was 14,916 in Rio de Janeiro, 4,141 in Salvador, and 2,979 in Manaus. A PrEP intervention that reaches 10% of MSM within 60 months would avert 338 infections in Rio de Janeiro (a 2.3% incidence reduction) by the end of year five (Fig. 1, top). Faster, more extensive uptake of PrEP increased the number of infections averted. If the intervention reached 10% of MSM within 24 months, more than twice as many infections (777) would be averted in Rio de Janeiro. An intervention reaching 60% of MSM within 24 months would avert 4,426 infections (a 29.7% incidence reduction).

For Salvador (Fig. 1, middle), the clinical impact of PrEP would vary from 94 infections averted and 2.3% incidence reduction (10% uptake within 60 months) to 1,254 infections averted and 30.3% incidence reduction (60% uptake within 24 months). For Manaus (Fig. 1, bottom), these same approaches would lead to 79 infections averted and 2.7% incidence reduction (10% uptake within 60 months) to 970 infections averted and 32.6% incidence reduction (60% uptake within 24 months).

In each city, outcomes varied more as a function of maximum uptake than time to attain that maximum; more infections would be averted by increasing uptake by one percent than by reducing the time to reach that uptake level by one month. For example, in Rio de Janeiro, compared with 10% uptake over 24 months, increasing uptake to 20% over 24 months would result in 755 additional infections averted. Reducing the time to maximum uptake would have less impact; compared with 10% uptake over 60 months, 10% uptake over 24 months would result in 439 additional infections averted.

Comparing cities, we found that the absolute number of infections averted for each level and speed of PrEP intervention would be greatest in Rio de Janeiro, reflecting its larger MSM population. In contrast, when focusing on the percent reduction in incidence at different uptake levels, values for Manaus would be slightly higher. With the most effective PrEP intervention evaluated (60% uptake within 24 months), the number of infections averted and percent incidence reductions for each city, are: Rio de Janeiro (4426; 29.7%), Salvador (1,254; 30.3%), and Manaus (970; 32.6%).

### Sensitivity analysis

**Fig. 2:**
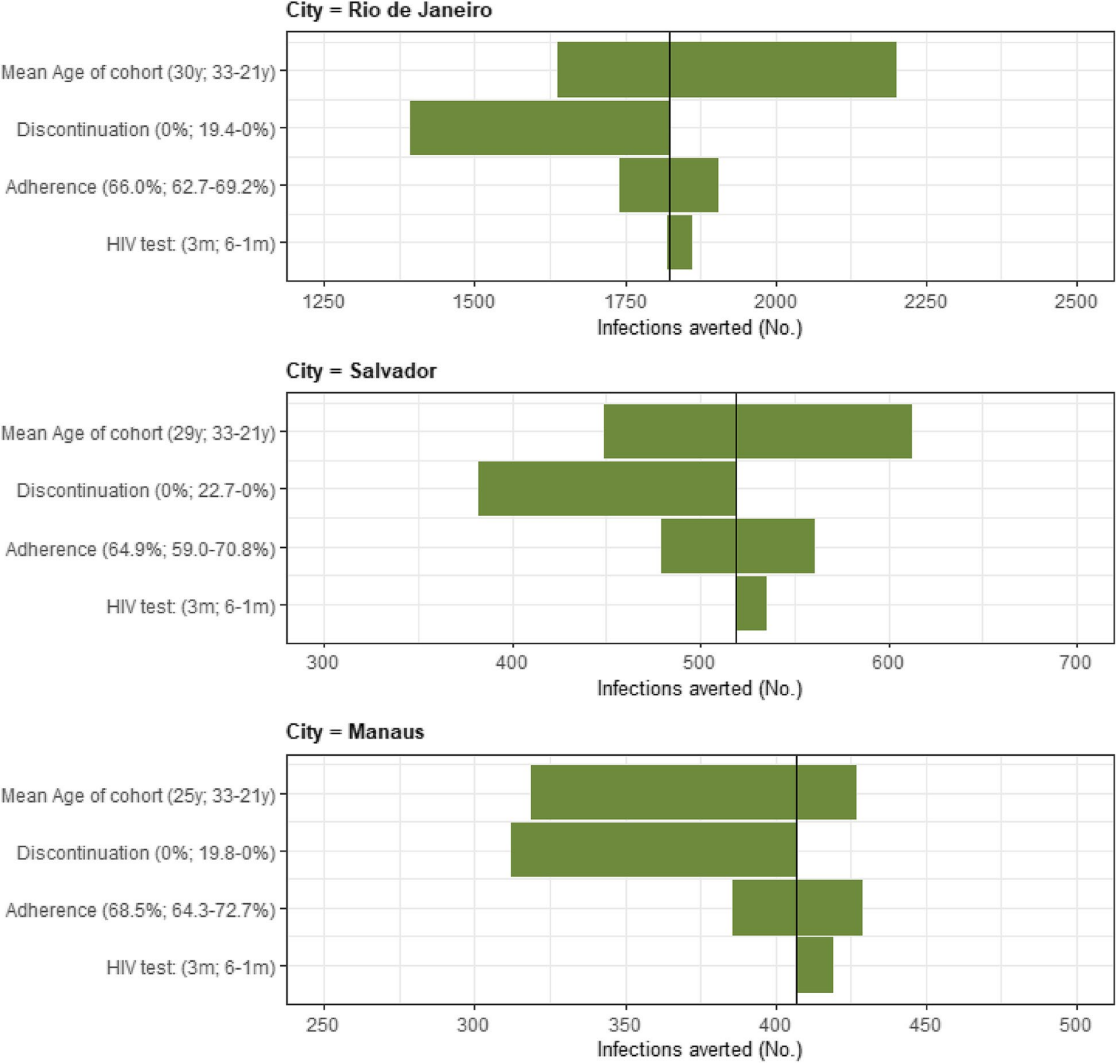
One-way sensitivity analysis results on number of averted HIV infections over five years for the PrEP intervention that considers 30% maximum uptake over 36 months for the following model parameters: adherence to oral PrEP, mean age of cohort, discontinuation from the PrEP intervention, and HIV testing frequency within the PrEP program. The x-axis represents number of infections averted over a five year time horizon and the y-axis is a categorical axis with the aforementioned model parameters as elements. The values written in parenthesis for each parameter represents the mean value (used in the base case) and range in which parameter values are varied, corresponding to lowest to highest number of infections averted, for each city. Top plot: Rio de Janeiro, middle: Salvador, bottom: Manaus. Footnote: y: years; m: months

For these analyses, we assumed a strategy of 30% maximum uptake achieved within 36 months; in the base case this led to 1,824, 519, and 407 infections averted in Rio de Janeiro, Salvador, and Manaus, respectively (Fig. 1). In all cities, the most influential parameter was the mean age of MSM at time of PrEP uptake. Decreasing the mean age of the cohort from the base case value of 30 years to 21 years averted 2,202 infections in Rio de Janeiro, 21% more than in the base case (Salvador: 18% and Manaus: 5%). When we assumed discontinuation as observed in ImPrEP, with 19.4% of participants discontinuing by the end of the first year in Rio de Janeiro, the number of averted infections decreased to 1,393, or 431 fewer than in the base case (Salvador: 137, Manaus: 95 fewer infections). In Rio de Janeiro, varying PrEP adherence within the ranges observed in ImPrEP, from a base case value of 66.0% to 69.2% increased averted infections to 1,905 (4% increase), while decreasing adherence to 62.7% decreased the number of averted infections by 5% to 1,742 (Fig. 2). Similarly for Salvador (and Manaus), we observed averted infections increase by 8% (Manaus: 5%) and decrease by 8% (Manaus: 5%) when the base case adherence value is changed to the upper and lower bound of the adherence ranges reported in ImPrEP for Salvador (Manaus). Additionally, we explored a scenario where adherence to PrEP was 95%, as a proxy for long acting PrEP, and found the number of averted infections increased by 41% compared to base case results. HIV testing frequency did not have a substantive impact on the results (Fig. 2).

**Fig. 3:**
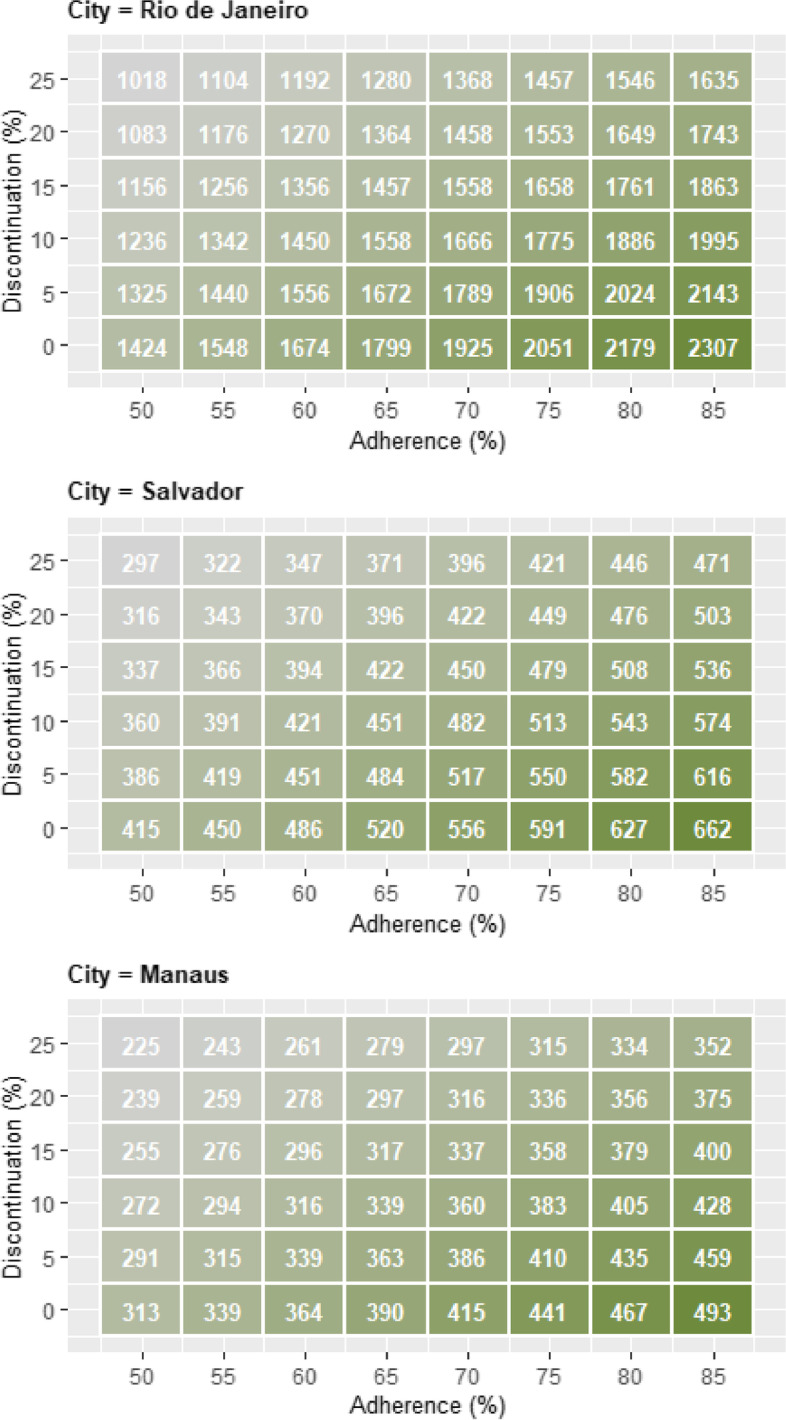
Two-way sensitivity analysis results for Rio de Janeiro (top), Salvador (middle), and Manaus (bottom) for the PrEP intervention that considers 30% maximum uptake over 36 months, when varying both adherence to PrEP and discontinuation rate of the PrEP intervention for the outcome number of averted HIV infections by the end of the fifth year

In two-way sensitivity analyses evaluating the joint effect of varying adherence and discontinuation rate (Fig. 3), again considering PrEP uptake of 30% over 36 months for the five year time horizon, we found that the impact of discontinuation rate was greatest when adherence was highest. Assuming 50% adherence, increasing the discontinuation rate from 0%/year to 25%/year reduced the number of averted infections from 1,424 to 1,018 in Rio de Janeiro. Assuming 85% adherence, changing the discontinuation rate from 0%/year to 25%/year reduced the number of averted infections from 2,307 to 1,635. With a discontinuation rate of 25%/year, adherence needed to be 75% to reach the same number of averted infections as when adherence was 50% with no discontinuation; findings in Salvador and Manaus were similar (Fig. 3).

## Discussion

HIV incidence among MSM in Brazil remains high [5], even though effective PrEP is now available free-of-charge to select populations [12]. We projected the potential impact of increasing PrEP uptake among MSM engaging in high-risk sexual behavior, focusing on three large cities. We found that reduction in HIV incidence after five years could range from 30 to 33% in each city, if PrEP uptake of 60% could be achieved within 24 months.

Adherence was a key determinant of the results. In Rio de Janeiro, for PrEP with 30% uptake in 36 months and no discontinuation, assuming lower adherence of 50%, as reported for some subpopulations of a local demonstration project in Brazil, led to a 22% decrease in averted HIV infections compared to the number of infections averted with a base case adherence of 66%. In contrast, if adherence was as high as 85% [9], a 26% increase in averted infections might be possible compared to the scenario with 66% adherence. Recent results of HIV Prevention Trials Network (HPTN) 083, a randomized double blind study that compared long acting injectable cabotegravir to daily oral PrEP showed that long acting PrEP was highly effective for preventing HIV in MSM [40]. In a scenario with PrEP adherence of 95%, as might be achieved with long-acting PrEP, the number of averted infections, compared to base case, increased by 41%.

Greater increases in averted infections could be achieved if PrEP strategies focused on younger MSM. We inferred the mean age of PrEP users as ~ 29 years from large surveys that used geosocial networking apps [21]. In sensitivity analysis, we found that if mean age of participants was 21 years, the number of averted infections compared to base case would increase by 21% in Rio de Janeiro. This is consistent with multiple epidemiologic findings that highlight the vulnerability of young MSM to HIV infection [21, 41, 42] and suggests that interventions focused on engaging young MSM in PrEP could have a major impact.

Our results are consistent with other studies showing that merely making PrEP available is not sufficient; engagement throughout the continuum of care is necessary to realize PrEP’s full potential [43–45]. A recent analysis of the population level impact of oral PrEP in Western Kenya highlighted how each step in the cascade of PrEP provision (uptake, adherence, retention, and reengagement) matters, in terms of averting HIV infections [43]. Similar to our study, the authors used local PrEP studies to inform their choice of model parameters and construct scenarios which assumed either 10% or 30% PrEP uptake, with results showing that losses along the cascade could decrease PrEP’s impact up to 98% [43]. Retention in PrEP care is also a challenge in many settings: recent estimates for oral PrEP uptake and use in a community based clinic in San Francisco/USA showed a discontinuation rate of 38% at 13 months since PrEP initiation [44], and a study conducted in multiple clinical sites in Chicago/USA found that only 43% of those initiating PrEP between 2012 and 2017 were retained in care for 12 months [45]. Among *ImPrEP* participants from the three countries (Brazil, Peru, and Mexico), attendance at the first two follow up visits within 120 days of PrEP initiation was achieved by 80% [46].

Though our model focused only on high-risk MSM, an eligible, target population for PrEP use as per Brazilian guidelines for PrEP provision through the National Health System, other modeling work has suggested that focusing uptake on high-risk groups would prevent more total infections [47, 48]. Guided by prior work and plausibility of real-world PrEP uptake [49], we assumed 60% as the highest achievable uptake, a finding that resonates with other modeling results showing that 50% uptake among populations at high risk of HIV infection might be cost-effective [48, 49]. Our results show, as expected, that the higher the uptake, the greater the percent incidence reduction. Nonetheless, though benchmark targets such as that proposed by UNAIDS might be helpful in setting goals, setting-specific targets for PrEP uptake should acknowledge the local HIV epidemic and will depend on appropriate estimates of the key populations at risk for HIV [23, 36].

A recent national analysis showed that among those initiating antiretroviral treatment as of 2015, 61% did so with a CD4 cell counts < 350 cells/mm [50]. In a more recent analysis from Rio de Janeiro that included those initiating treatment as of 2018, late treatment initiation (defined as CD4 cell count < 200/ul or AIDS-defining illness) was observed in 44.1% of participants [51]. Enhanced efforts to achieve earlier antiretroviral treatment initiation for people with HIV, combined with PrEP for those at high risk of HIV acquisition, would be a way to more substantially decrease HIV incidence [52]. However, the uptake and effectiveness of these approaches is limited by individual, network, community, and structural factors [53]. Societal stigma and discrimination towards sexual minorities may lead to avoidance of health services, including testing, lower likelihood of discussing and managing risks, as well as accessing and adhering to preventive or therapeutic interventions [53]. At the structural level, the past 10 years have witnessed a dismantling of health services both in treatment and prevention in Brazil [54]. Non-governmental organizations focusing on HIV prevention among MSM and transgender people have been defunded, removing spaces for community organization around themes of HIV prevention and testing, and for generating peer group support among communities [55]. Though biomedical treatment and prevention interventions are critical, addressing stigma, discrimination, and social exclusion are fundamental to achieving the coverage required to change the trajectory of the HIV epidemic among MSM [53].

This study has several limitations. We used a modeling framework to estimate the number of infections averted by year. We did not have specific incidence data for each city and we addressed data shortcomings by estimating the city- and age-specific incidence rates from the best available data. Additionally, we assumed, in the base case, no discontinuation from the PrEP intervention given the short time horizon used in the present analysis. This assumption allowed us to measure’s PrEP potential impact. However, a 2022 systematic review and meta-analysis estimated a discontinuation rate of 38% across different study timeframes, study designs, regions, age, and HIV incidence levels [56]. Interestingly, pooled discontinuation estimates from studies based in North America were significantly higher than other regions, with the South America region having the lowest estimate (8.9%, 95% confidence interval: 2.4% to 28.4%) [56]. The likely uptake of PrEP over time is unknown, so we investigated a wide range of levels, from 10 to 60%. The one global target of three million people on PrEP by 2020 has been recognized as unambitious as it translates into a PrEP uptake of only 10% [14]. Current UNAIDS strategy advocates a PrEP coverage of 50% for MSM at very high risk [14], a benchmark that we explored in this analysis. We have modeled PrEP uptake among eligible MSM thus implicitly assuming that those who discontinued remained at substantial risk, a reasonable assumption given that actual uptake is well below the pool of eligible MSM. Finally, we did not include the infrastructure or public information campaigns that might be needed to achieve higher uptake, though there is population access to health care providers within the structure of Brazil’s National Health System.

## Conclusions

Increased oral PrEP uptake in Brazil could substantially decrease HIV transmission, potentially by one third over five years. These results make a substantive case for the increased roll-out of daily PrEP in high incidence cities in Brazil. To increase PrEP’s impact on HIV incidence, efforts should focus on young MSM. Moreover, repeated encounters of PrEP users and potential PrEP users with health professionals offer multiple opportunities to remind individuals of the need to maintain adherence to PrEP. In considering structural barriers that affect access to health services by people belonging to sexual and gender minority groups in Brazil [57], these results highlight the importance of providing PrEP in non-stigmatizing environments to ensure low program discontinuation.

## Supplementary Information

**Additional file 1. **Indirect community benefit.

**Additional file 2. **CEPAC module: transmission network.

**Additional file 3. **Estimating HIV incidence rate by city.

**Additional file 4. **CEPAC modules: HIV natural history and Antiretroviral therapy regimens.

**Additional file 5. **Derivation of inputs for ART adherence, DTG-associated viral suppression, and late failure.

## Data Availability

Available from corresponding authors upon reasonable request. Details of CEPAC models are available at www.massgeneral.org/medicine/mpec/research/cpac-model

## Abbreviations

MSM: Men who have sex with men
HIV: Human immunodeficiency virus
PY: Person-years
PrEP: Pre-exposure prophylaxis
TDF/FTC: Tenofovir/emtricitabine
CEPAC: Cost effectiveness of preventing AIDS complications
iPrEx: Preexposure Prophylaxis Initiative trial
ImPrEP: Transnational PrEP implementation project in Latin America
PrEP Brasil: PrEP demonstration project in Brazil
HPTN: HIV Prevention Trials Network
UNAIDS: The joint United Nations programme on HIV/AIDS
