# Supplementary material for: Impact of pre-exposure prophylaxis uptake among gay, bisexual, and other men who have sex with men in urban centers in Brazil: a modeling study
Source: BMC Public Health. 2023 Jun 13;23:1128. doi: 10.1186/s12889-023-15994-0 (PMC10262537; doi:10.1186/s12889-023-15994-0)
Supplement: Supplementary file 1 — Additional file 1. Indirect community benefit. [file 12889_2023_15994_MOESM1_ESM.docx]

**Additional file 1: Indirect community benefit**

We define two benefits of PrEP: the direct individual benefit and the indirect community benefit. Both benefits result in a reduction in the force of infection (HIV incidence). The direct individual benefit will only be experienced by the susceptible individuals taking PrEP. Guided by drug efficacy and adherence to the drug, it reduces the risk of HIV infection for the duration PrEP is taken. The indirect community benefit of PrEP is the benefit generated from prevented HIV transmissions which otherwise would have occurred. Because there are fewer HIV-infected individuals when a PrEP intervention has been implemented, risk of infection will be lower for all susceptible individuals compared to the status quo. Hence, indirect community benefit will be experienced by everyone regardless of PrEP uptake status. We estimated the indirect community benefit of PrEP with a revised version of the method described in (1).

Each PrEP strategy (each intervention run) has a pre-simulation. Output of the pre-simulation is processed as described in (1) to measure ‘indirect community benefit’ specific to the PrEP strategy under consideration. The measured ‘indirect community benefit’ is applied as an exponential decay to the age-stratified status-quo incidence rate values in the main simulation. Hence, at the start of the main simulation, individuals not on PrEP are exposed to the same infection risk as those in a status-quo scenario. As simulation time progresses, individuals not on PrEP are exposed to a reduced risk compared to the status-quo arm because of the exponential decay factor. The reduction due to exponential decay is higher for better PrEP uptake strategies, i.e. strategies with either higher uptake and/or shorter time to reach the uptake level.
