# Supplementary material for: Impact of pre-exposure prophylaxis uptake among gay, bisexual, and other men who have sex with men in urban centers in Brazil: a modeling study
Source: BMC Public Health. 2023 Jun 13;23:1128. doi: 10.1186/s12889-023-15994-0 (PMC10262537; doi:10.1186/s12889-023-15994-0)
Supplement: Supplementary file 2 — Additional file 2. CEPAC module: transmission network. [file 12889_2023_15994_MOESM2_ESM.docx]

**Additional file 2: CEPAC module: transmission network**

Each HIV-infected individual in the cohort can transmit virus to susceptible individuals depending upon plasma HIV RNA level, HIV RNA level specific nominal transmission rates, and a transmission rate multiplier. HIV RNA level specific nominal transmission rate is highest during the acute phase of infection (62.56/100PY for individuals not on ART and 9.03/100PY for individuals on ART). With decreases in plasma HIV RNA level, the transmission rate decreases, and the rate is 0/100PY for HIV RNA < 20 copies/mL (2). The transmission multiplier is used in addition to the nominal transmission rates to calibrate the rate of transmission from HIV-infected to HIV-uninfected high-risk GBM. We only account for the transmissions from infected high-risk GBM to susceptible high-risk GBM and we estimated the proportion of such transmissions to be 70% of all transmissions from high-risk GBM (3).

The Figure below shows the modeled transmission of HIV with ‘S’, ‘I’ and ‘P’ representing susceptible, infected, and prevalent cases of HIV, that is, HIV infected individuals at model start; ‘HR’ and ‘LR’ stands for high-risk and low-risk, respectively. The solid black arrows entering $S_{HR}$will generate the total infection risk (monthly probability of infection). Individuals who take PrEP experience a lower infection risk according to efficacy of PrEP and the adherence level. This reduction in incidence is modeled by the ‘direct individual benefit’ (Appendix A). Moreover, due to PrEP roll-out in intervention strategy, the rate of growth of $I_{HR}$ and $I_{LR}$ will decrease, eventually reducing the risk of infection (HIV incidence) to $S_{HR}$. This reduction in incidence is modeled by ‘indirect community benefit’. In the Figure, there are two arrows going out from $I_{HR}$, the solid black arrow represents transmissions to high-risk GBM while the solid gray arrow represents transmissions to low-risk GBM from infected high-risk GBM. In this analysis, our focus is on GBM engaging in high-risk sexual behavior and on the impact of PrEP for this population, and as such we do not account for transmissions represented by the solid gray arrow.

**Figure: Transmission network modeled in CEPAC.** Model transmission network. ‘S’, ‘I’ and ‘P’ are susceptible, infected, and prevalent cases of HIV, ‘HR’ and ‘LR’ stands for high-risk and low-risk, respectively.

| 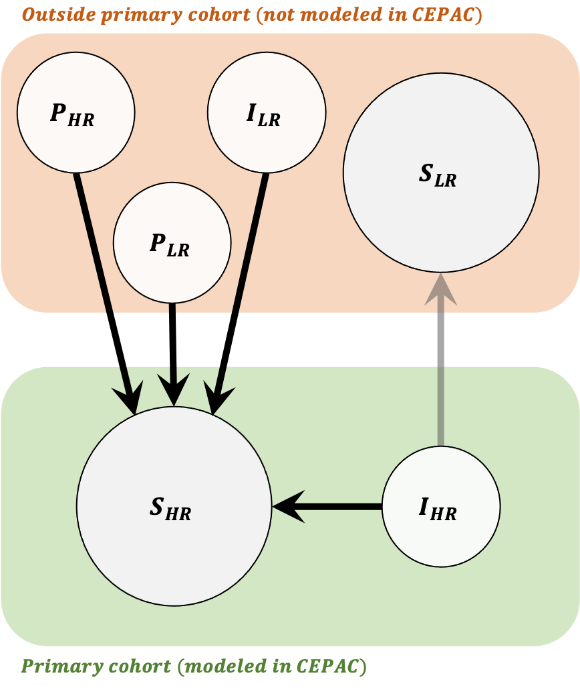 | \| $P_{HR}$ \| : HIV infected high-risk MSM  at model start \| \| --- \| --- \| \| $P_{LR}$ \| : HIV infected low-risk MSM  at model start \| \| $I_{LR}$ \| : HIV incident cases of low-risk MSM \| \| $S_{LR}$ \| : Low-risk HIV susceptible MSM \| \| $S_{HR}$ \| : High-risk HIV susceptible MSM \| \| $I_{HR}$ \| : HIV incident cases of high-risk MSM \| |
| --- | --- | --- | --- | --- | --- | --- | --- | --- | --- | --- | --- | --- | --- |
