# Supplementary material for: Impact of pre-exposure prophylaxis uptake among gay, bisexual, and other men who have sex with men in urban centers in Brazil: a modeling study
Source: BMC Public Health. 2023 Jun 13;23:1128. doi: 10.1186/s12889-023-15994-0 (PMC10262537; doi:10.1186/s12889-023-15994-0)

**Additional file 3: Estimating HIV incidence rate by city**

Using Rio de Janeiro as an example (a similar process was performed for the other cities), and using age-specific incidence rates from iPrEx (4) we defined incidence at age x (I_x_) as follows:

$$I_{x}=\left\{ \begin{matrix} \frac{4.3}{100PY} if x<40 \\ \frac{1}{100PY} if x\geq40 \end{matrix} \right.$$

We then fitted a Gaussian curve to the values defined by the equation above. The equation of the Gaussian curve is as follows,

$$\hat{I}(x;a, b,c)=a\cdot e^{-1\cdot\frac{\left( x-b \right)^{2}}{2c^{2}}}$$

Where, $\hat{I}(x;a, b,c)$ is the prediction of incidence value from Gaussian equation at the age $'x'$ and parameter values $'a', 'b', 'c'$. We find such values of the parameters $'a', 'b', 'c'$ in the curve fitting process that the mean squared error between the available incidence estimates and the Gaussian prediction of incidence is minimized. Therefore, our objective function is as follows,

$$\begin{matrix} arg min \\ a, b, c \end{matrix}\left( I_{x}-\hat{I}\left( x; a, b,c \right) \right)^{2}$$

Levenberg-Marquardt was the solution methodology used to solve the unconstrained optimization problem defined above. Let $a^{*}, b^{*}, c^{*}$, be the optimal parameter values, which were found to be as follows,

| $a^{*}=$ | $4.55$ |
| --- | --- |
| $b^{*}=$ | $21.14$ |
| $c^{*}=$ | $10.09$ |

Gaussian prediction of incidence rate values for each age group and published estimates are compared in Appendix Figure C.

**Figure. Gaussian curve fitting for incidence values.** Gaussian fitted estimates of HIV incidence rate as function of age used for model parametrization (Rio de Janeiro). PY: person-years


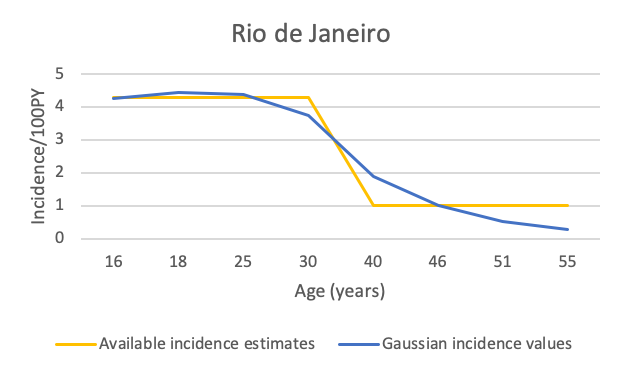

Supplement: Supplementary file 3 — Additional file 3. Estimating HIV incidence rate by city. [file 12889_2023_15994_MOESM3_ESM.docx]
