# Supplementary material for: Impact of pre-exposure prophylaxis uptake among gay, bisexual, and other men who have sex with men in urban centers in Brazil: a modeling study
Source: BMC Public Health. 2023 Jun 13;23:1128. doi: 10.1186/s12889-023-15994-0 (PMC10262537; doi:10.1186/s12889-023-15994-0)
Supplement: Supplementary file 4 — Additional file 4. CEPAC modules: HIV natural history and Antiretroviral therapy regimens. [file 12889_2023_15994_MOESM4_ESM.docx]

**Additional file 4: CEPAC modules: HIV natural history and Antiretroviral therapy regimens**

Upon infection, individuals enter the Disease module of CEPAC, are assigned an initial CD4 count and viral load, and experience disease progression according to user-specifiable HIV-related mortality, opportunistic infection (OI) risks, and propensity to respond to treatment (PTR). The Disease module of CEPAC simulates disease treatment and progression among those infected, dictated by CD4 and HIV RNA levels. Model users define a wide variety of screening and treatment parameters, including HIV screening practices (occasional or routine testing), OI prophylaxis and treatment, ART treatment, as well as HIV care practices regarding clinic visits and laboratory monitoring. Suppressive ART increases CD4 count and suppresses viral load, which leads to reduced probability of death through OIs or chronic AIDS.

*HIV natural history*

Mortality risks attributable to HIV and monthly opportunistic infection (OI) risks were derived from the HIV infected cohort of the Instituto Nacional de Infectologia Evandro Chagas, Fundação Oswaldo Cruz, in Rio de Janeiro using data from active participants during 2000 to 2010; OI prophylaxis policies were based on Brazil’s guidelines for care and treatment of people with HIV (5).

*Antiretroviral therapy regimens*

We used 2018 Brazil guidelines to inform ART parameters and laboratory monitoring, including genotype testing after treatment failure (5). First-line ART consisted of dolutegravir plus emtricitabine/tenofovir (DTG + TDF/FTC). Second-line ART consisted of ritonavir boosted darunavir (DRV/r) plus two nucleoside reverse transcriptase inhibitors (NRTIs). When patients failed either first line or second line ART, they were given another opportunity to resuppress on that same line. Last line ART consisted of DTG, a protease inhibitor (PI), and two NRTIs.
