# Supplementary material for: Impact of pre-exposure prophylaxis uptake among gay, bisexual, and other men who have sex with men in urban centers in Brazil: a modeling study
Source: BMC Public Health. 2023 Jun 13;23:1128. doi: 10.1186/s12889-023-15994-0 (PMC10262537; doi:10.1186/s12889-023-15994-0)
Supplement: Supplementary file 5 — Additional file 5. Derivation of inputs for ART adherence, DTG-associated viral suppression, and late failure. [file 12889_2023_15994_MOESM5_ESM.docx]

**Additional file 5: Derivation of inputs for ART adherence, DTG-associated viral suppression, and late failure**

In order to populate CEPAC heterogeneity and viral suppression inputs we estimated adherence distribution, 12-month viral suppression per regimen, the bivariate association between adherence and viral suppression status, and 24-month viral suppression (or probability of late failure) per regimen for Brazil.

*Adherence distribution:*

First, we conducted a literature search and performed a meta-analysis to estimate the proportion of individuals adherent to ART in Brazil (6-10). We used MetaXL to conduct the meta-analysis and estimated the prevalence of adherence as described by Barendregt et al (11). Briefly, we used double arcsine transformation and the method of inverse variance heterogeneity to weight the studies (12). We assessed the statistical heterogeneity using the I^2^ statistic and Cochran’s Q Chi^2^ test for heterogeneity, and data was presented using forest plots (Figure below).

Based on the meta-analysis, the proportion of individuals who are adherent was estimated as 72% (Figure below). The CEPAC input parameter for adherence is called the Propensity To Respond (PTR). PTR values are sampled for each simulated individual from a Gaussian distribution and parameters of this Gaussian distribution are required to be on logit scale. Hence, the mean of the Gaussian distribution for PTR is calculated as logit of the adherence value estimated from meta-analysis,

$$logit of adherence=ln\left( \frac{p}{1-p} \right)=0.958$$

And the standard deviation (SD) of the Gaussian distribution for PTR is calculated as follows,

$$variance of logit of adherence=\frac{1}{N*p*(1-p)}=\frac{1}{440*.72*(1-.72)}=0.01134$$

$$SD of logit=\sqrt{variance of logit} =\sqrt{.001134}=0.1065$$

The variance is inversely proportional to the sample size, therefore the higher the size the lower the variance. We selected the sample size (440) of the study used in determining the bivariate association between adherence and suppression (7).

Lastly, the model requires cutoff values, which were calculated as the inverse logit function. This value was obtained using the logistic CDF function in SAS [cdf('LOGISTIC',0.958030338, 0.106495894)], and cross checked with the value obtained from plogis function [plogis(0.958030338,0.106495894)] in R programming. Finally we used the cutoff value obtained from SAS function, which was 0.70089.

*12-month viral suppression:*

We obtained data related to viral suppression levels among those who receive DTG in Brazil. In order to calibrate the inputs to achieve the observed viral suppression levels of DTG in Brazil, we used data from Meireles et al. (85% at 12 months) (13).

We used the data from Costa et al. (7), to estimate the risk-ratio (RR) of suppression among the adherent compared to non-adherent cases at 12 months (~1.35). Using the overall suppression rate at 12 months and estimated RR, we calculated the 12-month viral suppression rate among adherent and non-adherent (0.96 and 0.67 respectively).

*Late failure on ART:*

In order to derive the probability of late failure we would need 24-month viral suppression data from Brazil. However, these data were not available. Therefore, we calculated the overall monthly probability of late virologic failure (i.e. not stratified by adherence status) from external clinical trials data (14) based on the following formula:

$$p(Late failure)=1-\left( \frac{pSupp(t_{2})}{pSupp(t_{1})} \right)^{1/(t_{2}-t_{1})}$$

Subsequently, the overall monthly probability of late failure (0.0035) was used to calculate the probability of late failure among adherent (0.0039) and non-adherent (0.0042) subjects.

To summarize, the model input values are as follows:

*Adherence:*

Adherence is defined as Gaussian distribution with mean of 0.9580 and standard deviation of 0.1065. The cut-off value was calculated from parameters of this Gaussian distribution as 0.70089.

*Probability of 12-month viral suppression:*

$$p\left( 12 month viral suppression \right)= \left\{ \begin{matrix} 0.6721 if sampled PTR\leq0.70089 (i.e. cutoff value) \\ 0.9622 if sampled PTR>0.70089 \end{matrix} \right.$$

*Probability of late failure:*

$$p\left( late virologic failure \right)= \left\{ \begin{matrix} 0.0041 if sampled PTR\leq0.70089 (i.e. cutoff value) \\ 0.0040 if sampled PTR>0.70089 \end{matrix} \right.$$

**Figure. Prevalence of adherence to ART**

Figure shows estimates of prevalence of adherence to ART published in four different studies (6-10). Values of the estimates and 95% CI are on the right hand side vertical axis and name of the first author of the respective study is on the left hand side vertical axis. In addition to the values and 95% CI, weights used for averageing adherence values are mentioned after the 95% CI. The elongated blue diamond at the bottom shows the result of the meta-analysis, i.e. 72% adherence.

CI: Confidence interval; Q: Q-value from Cochran’s Q Chi^2^ test for heterogeneity; I2: I^2^ statistic

**References**

1. Kazemian P, Costantini S, Neilan AM, Resch SC, Walensky RP, Weinstein MC, et al. A novel method to estimate the indirect community benefit of HIV interventions using a microsimulation model of HIV disease. J Biomed Inform. 2020;107:103475.

2. Attia S, Egger M, Muller M, Zwahlen M, Low N. Sexual transmission of HIV according to viral load and antiretroviral therapy: systematic review and meta-analysis. AIDS. 2009;23(11):1397-404.

3. Hansson D, Stromdahl S, Leung KY, Britton T. Introducing pre-exposure prophylaxis to prevent HIV acquisition among men who have sex with men in Sweden: insights from a mathematical pair formation model. BMJ Open. 2020;10(2):e033852.

4. Buchbinder SP, Glidden DV, Liu AY, McMahan V, Guanira JV, Mayer KH, et al. HIV pre-exposure prophylaxis in men who have sex with men and transgender women: a secondary analysis of a phase 3 randomised controlled efficacy trial. Lancet Infect Dis. 2014;14(6):468-75.

5. MS. Protocolo clínico e diretrizes terapêuticas para manejo da infecção pelo HIV em adultos. Available at <http://www.aids.gov.br/pt-br/pub/2013/protocolo-clinico-e-diretrizes-terapeuticas-para-manejo-da-infeccao-pelo-hiv-em-adultos>. Accessed on October 25 2021. 2018.

6. Costa JM, Torres TS, Coelho LE, Luz PM. Adherence to antiretroviral therapy for HIV/AIDS in Latin America and the Caribbean: Systematic review and meta-analysis. J Int AIDS Soc. 2018;21(1).

7. Costa JO, Ceccato M, Silveira MR, Bonolo PF, Reis EA, Acurcio FA. Effectiveness of antiretroviral therapy in the single-tablet regimen era. Rev Saude Publica. 2018;52:87.

8. Martin DA, Luz PM, Lake JE, Clark JL, Veloso VG, Moreira RI, et al. Improved virologic outcomes over time for HIV-infected patients on antiretroviral therapy in a cohort from Rio de Janeiro, 1997-2011. BMC Infect Dis. 2014;14:322.

9. De Boni RB, Shepherd BE, Grinsztejn B, Cesar C, Cortes C, Padgett D, et al. Substance Use and Adherence Among People Living with HIV/AIDS Receiving cART in Latin America. AIDS Behav. 2016;20(11):2692-9.

10. Miyada S, Garbin AJI, Gatto RCJ, Garbin CAS. Treatment adherence in patients living with HIV/AIDS assisted at a specialized facility in Brazil. Rev Soc Bras Med Trop. 2017;50(5):607-12.

11. Barendregt JJ, Doi SA, Lee YY, Norman RE, Vos T. Meta-analysis of prevalence. J Epidemiol Community Health. 2013;67(11):974-8.

12. Doi SA, Barendregt JJ, Khan S, Thalib L, Williams GM. Advances in the meta-analysis of heterogeneous clinical trials I: The inverse variance heterogeneity model. Contemp Clin Trials. 2015;45(Pt A):130-8.

13. Meireles MV, Pascom ARP, Duarte EC, McFarland W. Comparative effectiveness of first-line antiretroviral therapy: results from a large real-world cohort after the implementation of dolutegravir. AIDS. 2019;33(10):1663-8.

14. Sax PE, Pozniak A, Montes ML, Koenig E, DeJesus E, Stellbrink HJ, et al. Coformulated bictegravir, emtricitabine, and tenofovir alafenamide versus dolutegravir with emtricitabine and tenofovir alafenamide, for initial treatment of HIV-1 infection (GS-US-380-1490): a randomised, double-blind, multicentre, phase 3, non-inferiority trial. Lancet. 2017;390(10107):2073-82.
